# Grossesse sur cicatrice de césarienne: à propos d’un cas et revue de la littérature

**DOI:** 10.11604/pamj.2018.31.227.12905

**Published:** 2018-12-10

**Authors:** Meryem Belmajdoub, Sofia Jayi, Hikmat Chaara, Abdelilah Melhouf

**Affiliations:** 1 Centre Hospitalier Hassan II, Service de Gynécologie et Obstétrique II, Fès, Maroc

**Keywords:** Grossesse sur cicatrice de césarienne, grossesse ectopique, facteurs de risques, traitement conservateur, Cesarean-scar pregnancy, ectopic pregnancy, risk factors, conservative treatment

## Abstract

La grossesse sur cicatrice de césarienne est une forme rare de la grossesse ectopique qui peut engager le pronostic vital et fonctionnel de la patiente par hémorragie ou par rupture utérine précoce. Nous rapportons le cas d'une grossesse ectopique sur cicatrice de césarienne diagnostiquée à 7 semaines d'aménorrhée suite à des métrorragies chez une patiente de 23 ans, 3^ème^ geste 2^ème^ pare, porteuse d'un utérus bi-cicatriciel. Grâce à l'échographie endovaginale le diagnostic a été précoce et le traitement a été conservateur. A travers cette observation et la lumière d'une revue de la littérature, nous discuterons les caractéristiques diagnostiques et thérapeutiques de cette rare entité, dont la connaissance par les praticiens permet d'améliorer le pronostic.

## Abstract (en)

Cesarean-scar pregnancy is a rare form of the ectopic pregnancy which can be life-threatening or threaten patient's functional prognosis due to hemorrhage or early uterine rupture. We report the case of a 23-year old patient with ectopic pregnancy, gravida 3, para 2, with bi-scarred uterus who was diagnosed with cesarean-scar pregnancy due to metrorrhagias at 7 weeks of amenorrhea. Transvaginal ultrasound allowed early diagnosis and treatment was based on conservative therapy. This study and literature review aim to highlight the diagnostic and therapeutic features of this rare disorder whose knowledge can improve prognosis.

## Introduction

La grossesse sur cicatrice de césarienne est une grossesse ectopique de localisation cervico-isthmique et cicatricielle. Elle correspond à une implantation du sac gestationnel dans la cicatrice ou déhiscence de césarienne précédente. L'incidence est estimée entre 1/1800 et 1/2216 grossesses [1]. L'analyse de la littérature retrouve des séries limitées avec une augmentation du nombre de publications liées à ce sujet depuis les années 2000 puis surtout depuis 2006 [2]. La possibilité d'une grossesse sur cicatrice de césarienne est rarement suspectée, au profit des avortements en cours et des grossesses cervicales. Ainsi, la méconnaissance ou le diagnostic tardif pourraient être associés à des importantes complications telles la rupture utérine menant assez souvent à l'hystérectomie [3]. Nous présentons ici un cas de grossesse sur cicatrice de césarienne et à travers une revue de la littérature récente nous soulignerons les caractéristiques diagnostiques et thérapeutiques dont la bonne connaissance par les praticiens permet d'améliorer le pronostic de cette entité.

## Patient et observation

**Figure 1:**
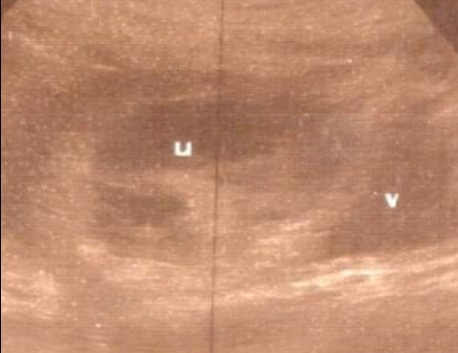
Sac gestationnel implanté sur la cicatrice de césarienne (image échographique sur une coupe sagittale)

**Figure 2:**
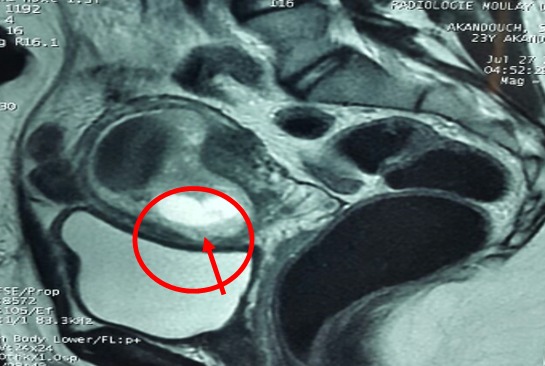
Grossesse sur cicatrice de césarienne avec épaisseur du myomètre < 2mm en regard (image IRM sur coupe sagittale)

**Figure 3:**
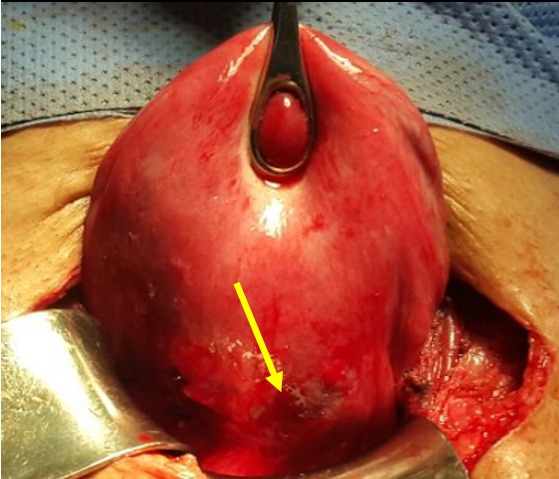
Utérus en rupture incomplète, la grossesse ectopique sur la cicatrice de césarienne est visible à travers la séreuse (image per opératoire)

**Figure 4:**
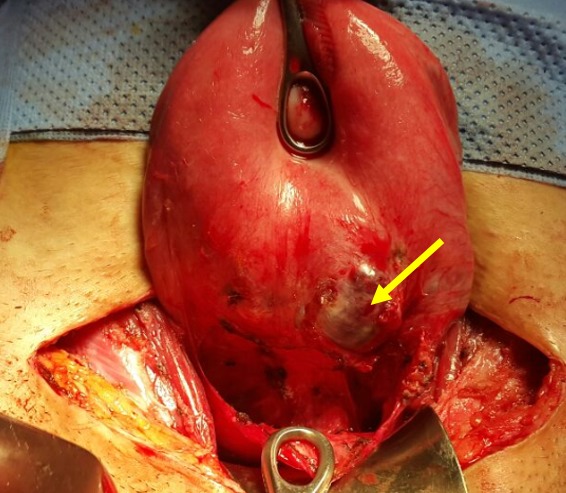
Après décollement vésico-utérin, la grossesse ectopique sur la cicatrice de césarienne est visible à travers la séreuse (image per opératoire)

**Figure 5:**
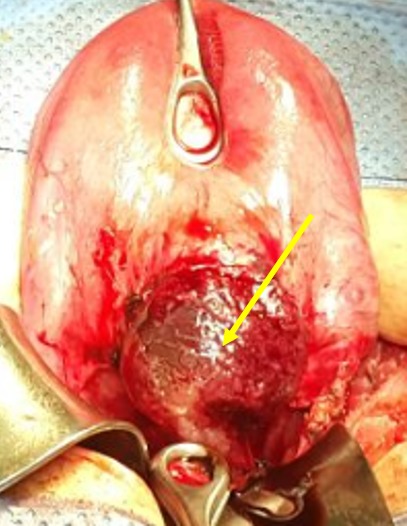
Incision en regard du site de la grossesse ectopique avec issu du produit de conception (image per opératoire)

**Figure 6:**
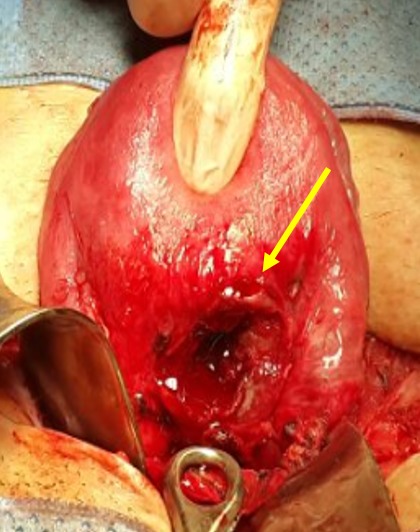
Aspect après résection complète du produit de conception (image per opératoire)

Mme AS âgée de 23 ans, 3^ème^ geste, 2^ème^ pare, porteuse d'un utérus doublement cicatriciel: la 1^ère^ césarienne était programmée pour dépassement du terme et la 2^ème^ l'était pour utérus cicatriciel avec macrosomie. Elle consulte aux urgences gynécologiques pour des douleurs pelviennes et métrorragies noirâtres de faible abondance évoluant depuis trois jours sur une aménorrhée de 7 semaines. A l'examen clinique l'état hémodynamique était stable, la palpation abdominale n'a pas retrouvé de sensibilité, l'examen au speculum a confirmé l'origine endo-utérine du saignement, et le toucher vaginal combiné au palper abdominal a objectivé un utérus légèrement augmenté de taille sans masse latéro-utérine ni signe d'irritation péritonéale. Une première échographie faite aux urgences avait objectivé: un utérus siège de 2 sacs gestationnels, le premier au niveau du fond à contenu hypo échogène (embryon en lyse), le 2^ème^ sac bas situé en regard du siège de l'ancienne cicatrice dont le trophoblaste semble s'insinuer au niveau de la cicatrice avec respect de la paroi vésicale, et un embryon dont la biométrie correspondait à l'âge gestationnel, avec activité cardiaque positive (Figure 1). L'IRM pelvienne était aussi en faveur d'une grossesse sur la cicatrice de césarienne avec une épaisseur myometriale en regard mesurée à moins de 2mm (Figure 2). A j+2 de son hospitalisation, la patiente a présenté une aggravation de la symptomatologie avec augmentation de l'intensité des douleurs pelviennes associée à des métrorragies de moyenne abondance ayant nécessité la réalisation d'une laparotomie. A l'exploration, on a noté la présence d'une tuméfaction bleutée isthmique gauche visible à travers le péritoine viscérale (Figure 3) dont l'incision après décollement abaissement de la vessie (Figure 4) a mis en évidence l'issue du produit de conception (Figure 5). Ainsi sa résection a été réalisée ainsi que celle de la cicatrice (Figure 6) avant de faire l'hystérorraphie. Les suites postopératoires étaient simples. Le taux de βHCG est revenu négatif au bout de 2 semaines.

## Discussion

La littérature récente suggère que la grossesse sur cicatrice de césarienne est plus fréquente qu'on ne le pensait précédemment. Son incidence est estimée entre 1/1800 et 1/2216 grossesse, et elle constitue 6,1% de toutes les grossesses extra-utérines avec une histoire d'au moins un accouchement par césarienne [3]. Les facteurs de risques incriminés sont similaires à ceux du placenta accreta: d'une part, le nombre de césariennes antérieures et de gestes endo-utérins (curetages, révision utérine manuelle), d'autre part, les techniques de fécondation in vitro (FIV) avec transfert d'embryon ont été aussi incriminées [1, 4]. D'un point de vue physiopathologique, un micro-defect de la cicatrice d'hystérotomie permettrait l'invasion du muscle utérin par le blastocyste: cette hypothèse est validée par la relation qui existe entre l'indication de césarienne antérieure et le risque de grossesse sur cicatrice, ces césariennes étant souvent programmées comme c'est le cas de notre patiente, le segment inférieur moins sollicité et moins mature ne permettrait pas une qualité optimale de cicatrisation et favoriserait l'implantation ectopique de l'œuf [5]. Les manifestations cliniques incluent les douleurs abdominales et les saignements, qui peuvent aller de simples spottings à une hémorragie mortelle [1]. Cependant la clinique peut être parfois asymptomatique, en effet, une étude de série avait retrouvé jusqu'à 40% de patientes ne manifestant ni douleur ni saignement vaginal [1]. D'où l'intérêt d'être attentif aux antécédents de la patiente. Le retard de diagnostic peut être à l'origine de la rupture utérine et une erreur diagnostique et une prise en charge comme fausse couche par curetage d'emblée pourraient entrainer une hémorragie massive [1]. Tout ceci souligne la grande importance du diagnostic rapide et précis [3] dans l'amélioration du pronostic vital et fonctionnel. L'échographie bidimensionnelle par voie endo-cavitaire est l'examen radiologique de première intention permettant de porter le diagnostic. Ce dernier repose sur les critères établis par Vial en 2000 [6] associant d'abord: un utérus vide; un canal cervical vide; l'existence sur une coupe sagittale de l'utérus, d'une disruption du sac gestationnel sur le mur utérin antérieur.

Il existe également des signes échographiques indirects, tels que la diminution de l'épaisseur du myomètre entre le sac gestationnel et la vessie qui reflète la profondeur de l'implantation et une hyper vascularisation péri-trophoblastique objectivée par le Doppler couleur ou énergie. En cas de doute diagnostique persistant après l'échographie, d'autres examens d'imagerie peuvent être réalisés comme le mode tridimensionnel échographique ou l'IRM qui permettent d'appréhender les rapports anatomiques en précisant la profondeur de l'invasion trophoblastique dans le myomètre, et l'atteinte potentielle de la séreuse ou de la vessie ainsi que la position exacte du sac gestationnel [7]. Les coupes sagittales et transversales en séquence pondérée T1 et T2 montrent clairement le sac ovulaire situé dans la paroi antérieure de l'utérus. Ceci permettrait de mieux apprécier le volume de la lésion et d'orienter les choix thérapeutiques [5, 7]. Si le diagnostic est évident à l'échographie bidimensionnelle, ces examens poussés ne sont pas recommandés [4]. Vu la rareté de cette situation il n'existe pas à l'heure actuelle de recommandations formelles concernant les modalités thérapeutiques. Le traitement considère l'âge gestationnel, les moyens thérapeutiques disponibles, le désir de fertilité ultérieure de la patiente, l'expérience de l'équipe thérapeutique, et les complications d'une thérapeutique de première ligne. Actuellement, le traitement, qu'il soit médical ou chirurgical, reste conservateur, sauf en cas d'échappement thérapeutique. Le traitement médical chez une patiente hémodynamiquement stable est envisageable pour beaucoup d'équipes. Il repose sur l'administration du méthotrexate par voie locale (injection in situ éventuellement sous écho ou coelioguidage) ou systémique ou l'association des deux à la dose de 1mg/kg [8]. Le taux de succès est similaire pour les deux voies d'administration et il est de l'ordre de 70 à 80%. Ce traitement nécessite une surveillance quotidienne de la décroissance des BHCG pendant la durée de l'hospitalisation puis une fois par semaine jusqu'à négativation, avec également une surveillance échographique jusqu'à disparition complète du sac ovulaire, avec un délai moyen nécessaire à la négativation des BHCG de 4 à 6 semaines. Les facteurs pronostics d'échec du traitement médical seraient les suivants: taux de BHCG supérieur à 10 000 UI/L, AG supérieur à 9 SA, présence d'une activité cardiaque fœtale à l'échographie, longueur crânio-caudale de l'embryon supérieure à 10mm en échographie.

**Figure 7:**
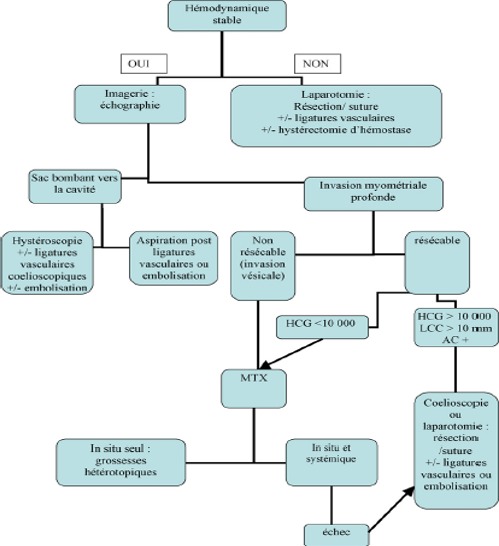
Arbre décisionnel: proposition de prise en charge thérapeutique en fonction des critères cliniques, biologiques et échographiques

Les différentes techniques chirurgicales sont généralement proposées en première intention aux patientes n'ayant plus de désir d'enfant, hémodynamiquement instable et/ou en cas d'échec du traitement médical. L'aspiration-curetage est à risque hémorragique et de rupture utérine, est contre-indiquée à l'aveugle. elle reste acceptable sous contrôle échographique en cas de sac gestationnel développé vers la cavité [5] et pour les grossesses de moins de 7 SA avec un myomètre sain antérieur (entre la grossesse et la séreuse utérine) supérieur à 3,5mm [9, 10]. La résection hystéroscopique est une méthode récemment décrite [11], elle nécessite une équipe entraînée pour réséquer la grossesse lorsqu'elle est accessible dans la cavité avec une coagulation efficace du pied vasculaire de la masse et présente des suites opératoires simples avec un retour à la normale trois fois plus rapide qu'après traitement médical. La laparoscopie et la laparotomie peuvent permettre une résection complète de la cicatrice et du tissu trophoblastique [1]. L'abord cœlioscopique tend à supplanter la laparotomie mais une grande expertise chirurgicale est nécessaire, garante d'une suture myometriale de qualité (donc solide en vue d'une grossesse ultérieure). La cœlioscopie permet enfin pour certains, de contrôler l'évacuation utérine par hystéroscopie ou simple curetage et d'associer une ligature artérielle préventive aux gestes utérins [12]. L'embolisation des artères utérines est une technique peu invasive qui permet un contrôle efficace de l'hémorragie, et peut être associée à toutes les méthodes thérapeutiques [13, 14]. Ainsi plusieurs procédés thérapeutiques ont été décrits dans la littérature en fonction des critères cliniques, biologiques et échographiques avec élaboration des arbres décisionnels (Figure 7) [15]. Dans notre cas, vue l'activité cardiaque positive, l'installation des métrorragies de moyenne abondance et la douleur pelvienne, on a opté pour la laparotomie avec résection complète de la cicatrice et du tissu trophoblastique et hystérorraphie.

Concernant le pronostic obstétrical: quelques grossesses ont été décrites après tout type de traitement conservateur [14], le risque de récidive existe, estimé à 5% [16]. Certaines équipes recommandent un délai de 12 à 24 mois entre la grossesse sur cicatrice de césarienne et une future grossesse [16]. Une évaluation de la cicatrice de césarienne est conseillée par certains auteurs avant d'envisager une autre grossesse. Certaines équipes utilisent l'hystérosonographie pour évaluer la cicatrice de césarienne [16, 17] à la recherche d'un defect au niveau de la cicatrice, pour d'autres l'échographie présente une sensibilité de 87% et une spécificité de 100% dans le diagnostic de ce defect utérin au cours de la grossesse [17]. Les auteurs préconisent alors de réaliser une échographie précoce lors d'une grossesse ultérieure afin de vérifier la localisation intra-utérine du sac gestationnel [17]. La voie d'accouchement privilégiée serait alors la césarienne programmée dès que la maturation pulmonaire est acceptable, au vu du risque augmenté de rupture utérine. Ils recommandent alors de procéder à une césarienne prophylactique vers 37 SA et d'anticiper les risques d'hémorragie du post-partum en ayant accès à un centre d'embolisation [14]. Concernant notre patiente, suite à un oubli de pilule, elle est tombée enceinte 14 mois après, le suivi échographique depuis le début de la grossesse n'a objectivé aucun signe de déhiscence et une césarienne prophylactique a été réalisé à 37 SA avec à l'exploration présence d'un segment inférieur trop fin sans aucun signe de déhiscence avec extraction d'un nouveau-né du sexe féminin avec poids à la naissance 3200g, les suites post opératoires étaient sans particularités.

## Conclusion

La survenue d'une grossesse sur cicatrice de césarienne n'est plus un évènement exceptionnel. Elle fait à présent partie intégrante des complications à long terme des césariennes. Elle peut être classée au même niveau de gravité que le placenta accreta. L'intérêt d'un diagnostic précoce réside dans la possibilité de choisir une thérapeutique adaptée en fonction du contexte clinique, des données radiologiques, du plateau technique et du désir de la patiente. Ceci pourrait limiter les complications hémorragiques graves qui très souvent s'accompagnent d'hystérectomie totale compromettant ainsi la fertilité ultérieure de la patiente quand le décès maternel a pu être évité.

## Conflits d’intérêts

Les auteurs ne déclarent aucun conflit d'intérêts.

## Remerciements

Nos remerciements s'adressent à tous nos maitres et à toute l'équipe du Service des Maladies Infectieuses et Tropicales et aux patients dont les dossiers ont été retenus pour cette analyse.
